# Hyperactivity of the default mode network in schizophrenia and free energy: A dialogue between Freudian theory of psychosis and neuroscience

**DOI:** 10.3389/fnhum.2022.956831

**Published:** 2022-12-14

**Authors:** Jessica Tran The, Jean-Philippe Ansermet, Pierre J. Magistretti, Francois Ansermet

**Affiliations:** ^1^INSERM U1077 Neuropsychologie et Imagerie de la Mémoire Humaine, Caen, France; ^2^Centre Hospitalier Universitaire de Caen, Caen, France; ^3^Université de Caen Normandie, Caen, France; ^4^Ecole Pratique des Hautes Etudes, Université Paris Sciences et Lettres, Paris, France; ^5^Agalma Foundation Geneva, Geneva, Switzerland; ^6^Cyceron, Caen, France; ^7^École Polytechnique Fédérale de Lausanne, Lausanne, Switzerland; ^8^Division of Biological and Environmental Sciences and Engineering (BESE), King Abdullah University of Science and Technology, Thuwal, Saudi Arabia; ^9^Brain Mind Institute, Swiss Federal Institute of Technology Lausanne, Lausanne, Switzerland; ^10^Département de Psychiatrie, Faculté de Médecine, Université de Genève, Geneva, Switzerland

**Keywords:** default network, schizophrenia, trace reassociation, psychoanalysis, free energy

## Abstract

The economic conceptualization of Freudian metapsychology, based on an energetics model of the psyche's workings, offers remarkable commonalities with some recent discoveries in neuroscience, notably in the field of neuroenergetics. The pattern of cerebral activity at resting state and the identification of a *default mode network* (DMN), a network of areas whose activity is detectable at baseline conditions by neuroimaging techniques, offers a promising field of research in the dialogue between psychoanalysis and neuroscience. In this article we study one significant clinical application of this interdisciplinary dialogue by looking at the role of the DMN in the psychopathology of schizophrenia. Anomalies in the functioning of the DMN have been observed in schizophrenia. Studies have evidenced the existence of hyperactivity in this network in schizophrenia patients, particularly among those for whom a positive symptomatology is dominant. These data are particularly interesting when considered from the perspective of the psychoanalytic understanding of the positive symptoms of psychosis, most notably the Freudian hypothesis of delusions as an “attempt at recovery.” Combining the data from research in neuroimaging of schizophrenia patients with the Freudian hypothesis, we propose considering the hyperactivity of the DMN as a consequence of a process of massive reassociation of traces occurring in schizophrenia. This is a process that may constitute an attempt at minimizing the excess of free energy present in psychosis. Modern models of active inference and the free energy principle (FEP) may shed some light on these processes.

## Introduction

The economic conceptualization of Freudian metapsychology, which is based on an energetics model of the psyche's workings, offers remarkable commonalities with some recent advances in neuroscience, notably discoveries in the field of neuroenergetics (Friston et al., 2006; Tran The et al., 2018; Cieri and Esposito, 2019). As early as 1895, in his *Project for a Scientific Psychology* (1895a), Freud's intention had been to base his understanding of normal and pathological psychic processes on a description of brain function. At that time, he had postulated the existence of a fundamental tendency of the nervous system to reduce all quantities of neuronal excitation: “the principle of neuronal inertia.” This speculative proposal on brain function rested on a concept of “quantity” of neuronal energy or intracerebral excitation a theory influenced both by the physicalism of the Helmholtz school (Helmholtz, 1882), and by Oswald's energetics model. Oswald stated that: “…psychological phenomena can be construed as energetic phenomena, and interpreted as such just as well as any other phenomena” (Oswald, 1891). This desire to apply the overarching principles of thermodynamic physics to living organisms, and more specifically to the nervous system, supports the view that the father of psychoanalysis was following in the tradition of the Helmholtz School and its director, Brücke.

Yet, Freud will distance himself from this ambition to explain the psychological processes by reference to the physiology of the nervous system. He did not proceed with the publication of the *Project* manuscript, which remained unfinished, because of the limitations of biological knowledge at that time:

“[I am] not at all inclined to leave the psychology hanging in the air without an organic basis. But apart from this conviction I do not know how to go on, neither theoretically nor therapeutically, and therefore must behave as if only the psychological were under consideration.” (Freud, 1898, p. 326)

Thus, from 1897, with *The Interpretation of Dreams* (Freud, 1900), Freud abandoned his references to anatomy and adopted an exclusively psychological vocabulary. It is at that point that he introduced the fundamental concepts of his metapsychology or theory of the unconscious psychic processes. He did this, however, without completely forgoing his energetics concept of the psychic processes, since the quantity of psychic energy becomes the concept of *libido*.

“I refer to the concept that in mental functions something is to be distinguished – a quota of effect or sum of excitation – which possesses all the characteristics of a quantity (though we have no means of measuring it), which is capable of increase, diminution, displacement and discharge, and which is spread over the memory-traces of ideas somewhat as an electric charge is spread over the surface of a body.” (Freud, 1894, p. 60)

Although Freud had renounced his hope of being able to measure that quantity of energy and link it to processes in the brain (Rapaport, 1958), the psychoanalytic concept of the psychic processes in terms of energy shows some surprising commonalities with the field of neuroenergetics. This new field of research has been opened up by the technical advances made in neuroscience, notably in the area of neuroimaging. In 1920, Freud himself had imagined the feasibility of such an interdisciplinary dialogue:

“Biology is truly a land of unlimited possibilities. We may expect it to give us the most surprising information and we cannot guess what answers it will return in a few dozen years to the questions we have put to it.” (Freud, 1920, p. 60)

If psychoanalysis and neuroscience remain two fields with their own epistemological foundations, today these two fields do seem to have found some common ground in an energetics concept of the living organism (Ansermet and Magistretti, 2010). From a strictly biological perspective energetics has become one of the leading ideas. Recent neuroscientific research has brought to light important data relating to what could be qualified as an intrinsic activity belonging to living organisms. In particular, the developments of recent techniques in neuroimaging such as positive emission tomography (PET) or functional magnetic resonance imaging (fMRI), has offered scientists the possibility to observe regions of the brain activated during the performing of specific tasks–be they motor, sensorial or cognitive tasks.

Since the 2000's, some scientists have started to take an interest in the background brain activity. This is activity that takes place during states of rest when no task or specific behavior is taking place. In particular, Marcus Raichle has demonstrated that, at rest (i.e., not performing any particular activity), some areas of the brain are more active than during the performing of a specific task (Raichle and Gusnard, 2002). There exists therefore an activity intrinsic to the brain, a background activity, one that is particularly costly in energy. This cerebral network, active during *resting state* and called *default mode network* (DMN), presents a promising field of research for the dialogue between psychoanalysis and neuroscience around a concept of the living organism centered on energetics. And we can try to throw light on the Freudian energetics approach to psychopathological processes with these recent neuroscientific data.

In the *Project* (1895a), Freud's energetics concept was initially principally motivated by his clinical observations of neurotic patients. He stated that his concept of quantity “is derived directly from pathological clinical observation” and that in patients with neuroses “the quantitative characteristic emerges more plainly than in the normal.” (Freud, 1895, p. 295).

In his *Studies on Hysteria* (1895d), Freud put forward a theory of the psychopathology of hysteria as being a consequence of the increase in the quantity of energy (psychic excitation or *quantum of affect*). This quantity of excitation was then discharged by somatic means, through the expression of symptoms of conversion (Freud and Breuer, 1895). In a more general way, as his texts on the neuro-psychoses of defense demonstrate (Freud, 1894), right from the start Freud understood the different psychic pathologies as being linked to pathological alterations in quantities of psychic energy (later termed libido). These alterations could be linked to acquired factors (for example subsequent to trauma) (Freud and Breuer, 1895), as well as hereditary factors, insofar as in certain subjects heredity “Facilitates and increases the pathological affect” (Freud, 1896b, p. 162) in the same way as “…a multiplier in an electric circuit, which exaggerates the visible deviation of the needle,” (Freud, 1896a, p. 147).

Subsequently, during his study of the Schreber case, Freud extended this hypothesis for a libidinal etiology of psychic pathologies to the treatment of psychosis. At that moment he proposed an innovative theory of this illness, based on a diachronic perspective, by arguing that psychosis stemmed from a libidinal process in two phases. The initial process of psychosis consisted in massive withdrawal of the libido (or quantity of psychic energy) into the ego and the body itself (Freud, 1914). This was the first phase of the psychotic process, dominated by significant alteration in the perception of the body. The *positive* symptoms, and importantly the patients' delusional constructions, belong to the second phase of psychosis, and came as an attempt to reinvest the quantity of energy externally, on objects and delusional representations. Delusion was thus, in Freud's view, an “attempt at recovery” (Freud, 1911).

This Freudian perspective of a libidinal etiology for psychosis, which fits into the framework of a concept of pathologies of the psyche based on energetics (and the “economics” view of the metapsychology), can be integrated into neuroscientific discoveries about DMN, and into recent integrated models of brain function. In the *Project*, Freud introduced energy quantities based on language of Helmoltzian thermodynamics (Tran The et al., 2018). Nowadays, a new conceptual framework has emerged from Bayesian statistics and information theory (Parr et al., 2022). Thus, Friston and coworkers proposed a dialog between cognitive science, neuroscience and psychoanalysis, based on the notion of Active Inference which is rendered quantitative thanks to the principle of minimization of a free energy (Carhart-Harris and Friston, 2019).

Karl Friston and others have proposed introducing a new model for a dialogue between psychoanalysis and neuroenergetics based on the FEP. The FEP proposes that living organisms, as organized systems in a state of equilibrium with their environment, need to minimize their free energy, which is to say they need to resist the natural tendency to disorder (Friston, 2009; Ramstead et al., 2018). As has been suggested by Mark Solms, this minimizing of the free energy is similar to a homeostatic understanding of the regulation of the physiological states, which would be one of the most basic functions of consciousness-based somatic and affective states (Solms, 2013; Solms and Friston, 2018). In Solms' words, “…the long-sought mechanism of consciousness is to be found in an extended form of homeostasis” (Solms, 2019). By integrating the idea of free energy into a Bayesian approach to cognition, where the brain is understood as a machine for inference (Helmholtz, 1866; Gregory, 1968; Dayan et al., 1995), this model proposes that the brain uses internal hierarchical models to predict sensorial inputs. The hypothesis is that neuronal activity and synaptic connections try to minimize the ensuing prediction-error resulting from the inference processes (or try to minimize the free energy) (Carhart-Harris and Friston, 2010; Parr et al., 2022). But the FEP does not only constitute an epistemological focus in the interdisciplinary dialogue. It also opens onto important clinical applications in the field of psychopathology (Rabeyron, 2022).

From this standpoint, we will try to open a dialogue between Freud's view of delusion as an “attempt at recovery” and recent data and models coming from the neurosciences, which have been obtained through to the techniques of neuroimaging. The data attest to the existence, in some psychiatric illnesses, of anomalies in the neuroenergetic activity of the brain. In this present article, we focus particularly on the literature that evidences the existence of hyperactivity of the DMN in schizophrenia, that is to say: an abnormally increased activity in energetic terms observed in the cerebral activity at rest, in patients with schizophrenia. In this article we propose using an interdisciplinary approach to shed light on these data, through the prism of a dialogue between psychoanalysis and neuroscience. To do this we will make reference to the Freudian hypothesis of delusion as an “attempt at recovery,” and integrate these elements to the FEP model.

To this end, we will first give an overview of the discovery of the DMN and the hypotheses relative to the DMN's possible functions. We will identify some common ground between the DMN and the binding function of the ego in Freudian theory, into the framework of the FEP. Then we will review the anomalies in this network observed in schizophrenia. Next, having reviewed the Freudian concept of the positive symptoms of psychosis–notably his hypothesis of delusion as an attempt at recovery– we will bring together the data on the hyperactivity of the DMN in patients with schizophrenia and these Freudian hypotheses. Through this reflection, we propose interpreting the hyperactivity in the DMN as consequent to a massive process of reassociation of traces occurring in schizophrenia. The FEP perspective might be invoked to clarify the dynamics of this particular process. In this light, delusion could constitute an attempt at binding the free energy through the reassociation of traces, so as to minimize entropy and try to re-establish an allostatic equilibrium within the psyche.

## The discovery of cerebral activity during resting state and the default mode network

Initial research in neuroenergetics, undertaken by Louis Sokoloff and Seymour Kety in the 1950's, already revealed that the cost of internal functional activity represented a significant part of the total energy expenditure of cerebral activity (Sokoloff et al., 1955). While the average adult brain represents ~2% of the total body weight, it nevertheless uses 20–25 % of the organism's total energy consumption–and that, even during resting state (Sokoloff et al., 1955). The performing of tasks therefore, has only a minimal incidence on the massive energy consumption of the brain, producing only a rise of barely 5–10 % of the total consumption (Fox and Raichle, 2007). Marcus Raichle, in the 1990's, began to envisage this baseline activity as a promising field for research (Raichle, 2006), and a source of new data for the understanding of certain psychopathological processes (Fox and Raichle, 2007).

The development of different imaging techniques, such as PET and fMRI made it possible to identify a network of active areas when cognitive tasks were not being performed: the medial prefrontal cortex, posterior cingulate cortex, the inferior parietal lobules and the hippocampal regions (Raichle et al., 2001; Andrews-Hanna et al., 2014). The work done by Raichle, on the basis of the data furnished by the study of neuroimaging, led to the description and theorisation of the DMN. A default mode that attests to the existence of neural and metabolic activity that is intrinsic to the brain, and which is not linked either to a particular environmental stimulus, nor to the performing of a specific motor or cognitive task (Buckner et al., 2008).

In the state of consciousness termed random episodic silent thinking (REST)–during which the individual processes corporeal information (somatosensory and vegetative), and during which the individual also free associates ideas, emotions, and mental images connected to the past or to future planning–the attentional system is not solicited, and it is the DMN that is active. This is the spontaneous brain activity generated in the absence of any cognitive task (Raichle, 2001; Buckner et al., 2008; Cieri and Esposito, 2018, 2019).

Raichle proposed defining this default mode, or baseline state, as the brain activity that goes with a subjective state of rest, when the subject presents a behavior characterized by calmness, eyes closed (or open but with no specific focus), in a state of consciousness filled with thoughts that are independent of external stimuli, a state that might be described as “daydreaming” (Raichle, 2010). Based on the data from functional imaging, the brain is in fact never really at rest, insofar as the consumption of energy linked to its intrinsic activity, independent of any action or perception of external stimuli, is extremely high. This baseline activity can be observed even under general anesthesia, or in the first stages of sleep. Furthermore, one of the major discoveries made by Raichle and his team was to bring to light a diminution of the default mode activity during the performing of tasks that require directed attention. That is to say that, certain brain regions active at rest deactivate during the performing of a conscious goal-directed task (Shulman et al., 1997; Raichle, 2010; Passow et al., 2015).

This reciprocal deactivating of the DMN and the networks linked to the attention system, such as the dorsal attention network (DAN), which appears to be anticorrelated (Fransson, 2005), involves an opposition between an event-related brain activity and spontaneous brain activity (evoked/spontaneous). The latching mechanism between the two networks may demonstrate a change in attention that switches from an internal self-referential state to an outwardly focused state (Raichle, 2001), with a shift of attention between the internal and the external. The attention system, converse to the DMN, is activated when cognition is directed outwards and the opposite is true for the DMN (Buckner et al., 2008). However, recent research indicates that the attention system is not a unified one. Besides the DAN, the salience network is also active during the performing of goal-directed tasks (Corbetta and Shulman, 2002; Fox et al., 2006; Carhart-Harris and Friston, 2010; Esposito et al., 2018).

The mechanisms that contribute to the deactivation of the DMN during the execution of a task that is demanding at a cognitive level, and involves focused attention on the environment, are still poorly understood. There are however some studies that have brought to light a significant co-activation of a third network during the “switch” between the activity of the DMN and that of the attention system (Sridharan et al., 2008). The salience network (a network comprising the right insular cortex and the anterior cingulate cortex) is responsible for switching between the default mode network and the central executive network (Goulden et al., 2014). Scientists have therefore concluded that the insular cortex plays a key role in this switch over between the internal cerebral activity and activity that is directed toward the environment; and is probably linked to the importance of the insula in distinguishing between endogenous processes and exteroceptive perceptions (Craig, 2002; Tran The et al., 2020).

## Hypotheses relating to the functions of the default *mode*

To better understand the mechanisms of the DMN, Raichle initially based his thinking on what was already known regarding the behavioral and cognitive functions associated with the main anatomical subdivisions of the baseline state network, that is: the ventromedial prefrontal cortex, the dorsomedial prefrontal cortex, and the posterior cingulate cortex, the adjacent precuneus, the lateral parietal cortex (Raichle, 2015).

The ventromedial prefrontal cortex in particular is involved in the processing of sensory information from the environment, coming from the orbitofrontal cortex, and then transmitted to structures such as the hippocampus and the amygdala (Raichle, 2015). Data from patients having suffered brain damage have highlighted the importance of this area in the control of social behavior, mood, and motivation (Damasio et al., 1994). Furthermore, studies of imaging performed on healthy individuals have shown that the emotional state (as well as the anxiety levels) of the subject has a direct effect on the levels of activity in the ventromedial prefrontal cortex within the baseline state's network (Park and Moghaddam, 2017). An increase in the activity of the areas of the ventromedial prefrontal cortex can also be observed during disturbances in the body's homeostasis, for example during a state of hypoglycaemia (Teves et al., 2004). It can be supposed therefore that the baseline state, in that its network involves the activation of the ventromedial prefrontal cortex, may be involved in the processing of emotions and disruption to homeostasis. The dorsomedial prefrontal cortex, although it is adjacent to the ventromedial prefrontal cortex, is involved more specifically in the perception of the self, and in the processing of emotions that accompany that perception of the self (Raichle, 2015).

The posterior cingulate cortex and the medial precuneus make up another area, notably associated with lateral parietal elements of the DMN, that is particularly active during the baseline state. A study made by Vincent et al. has brought to light that these specific areas of the parietal cortex are involved in episodic memory. Studies of functional imaging attest to their systematic activation when a memorizing task has successfully been performed following learning (Vincent et al., 2006). This study has also contributed to highlighting the existence of a neuronal network linking these parietal areas to the hippocampus, all of these networks being activated when the subject is at rest in the absence of a task or explicit mnesic stimulation. This activity is particularly increased if the resting state is consecutive to activities involving memory or the recall of elements previously memorized (Shannon et al., 2013).

More generally, the study of these data on the functioning of the different anatomical areas belonging to the DMN indicate that it are linked to thought processes such as daydreaming (Buckner et al., 2008), self-referential mental activity (for example attention focused on emotional or physical states), the processing of autobiographical memories, or thoughts about the future (Doucet et al., 2011; Dor-Ziderman et al., 2013), and theory of mind (Carhart-Harris and Friston, 2019). This has led scientist to hypothesis that the DMN could be the neurobiological seat of the ‘self' (Andrews-Hanna, 2012; Cieri and Esposito, 2019).

In so far as that the activity of the default mode seems to correlate strongly with the recollection of autobiographical memories (Vincent et al., 2006; Buckner et al., 2008; Doucet et al., 2011), this cerebral network could constitute one of the biological foundations of what Damasio has defined as our awareness of an autobiographical self. This autobiographical self is underpinned by old memories, the stability of which guarantees the grounds for our sense of identity regardless of the continuous changes to which we are subject (Damasio, 2000). From the perspective of *embodied cognition*, the sense of our autobiographical self and identity is also intimately dependent on certain representations linked to out physical cartography, to the mental image of our own body, within certain areas of the brain. The neuronal mechanisms that generate this representation can be seen as the foundation for a kind of stability of the organism–which Damasio terms the “proto-self”–that is at the root of our sense of self. It is on the basis of this stable representation of the body–an anchor from the point of view of an invariable reference through time–that the sense of an autobiographical self will subsequently develop. This is an autobiographical self from which stems our sense of identity regardless of the continuous changes that shape us and that:

…might give the brain a natural means to generate the singular and stable reference we call self […] biologically speaking, grounded on a collection of nonconscious neural patterns standing for the part of the organism we call the body proper. (Damasio, 2000, pp. 134-135)

Within the DMN certain areas may be involved in the integration of external stimuli, such as interoceptive physical sensation, as well as information coming from the environment, for example visual and auditory perceptions (Phan et al., 2002). In particular, the interaction between the DMN and the insula may contribute to this integration between the external and interoceptive sensations (Molnar-Szakacs and Uddin, 2013; Cieri and Esposito, 2019). Research on the anterior insula undertaken by Craig has made it possible to characterize the neurobiological foundations of this “interoceptive” sense, which furnishes the organism with a representation in real time of the state of its various organs. Indeed, even in the absence of external stimuli from the environment, the brain constantly processes endogenous perceptions coming from the viscera, the muscles, and the joints, as well as the different concentrations of hormones in the blood stream, which are constantly being detected by the nervous system. Information coming from the body is initially processed in the medial and posterior areas of the insula then, in a second phase, integrated within the anterior insula (Craig, 2002). The integration between the perception of exteroceptive experiences and the representations coming from the interoceptive endogenous perceptions, in so far as that it is a process staggered in time in relation to the initial experience (and which therefore does not necessarily occur during the initial perception of the external stimuli), could thus be part of the cerebral activity of the brain at resting state (Ansermet and Magistretti, 2010).

These observations have in turn led some scientist to draw a parallel between the function of the self, notably in its Freudian definition, and the DMN (Carhart-Harris and Friston, 2010; Cieri and Esposito, 2019; Cieri, 2022). This analogy is based on evidence that this network has been linked to spontaneous cognition and self-referential activity that not only involves remembrance of the past and projection into the future but also the processing of interoceptive content. Indeed, the psychoanalytic definition of an example of the psychic self, involves an important physical and interoceptive dimension: according to Freud, the primary formation of the ego, in the phase of “primary narcissism,” would occur with the libidinal cathexis of the subject's own body, when all the psychic energy unifies and organizes itself taking the ego as object (Freud, 1914). The ego would then become the object of the drive–the sexual drives finding their origin in excitation coming from the bodily organs. Thus, according to Cieri, in so far as in Freudian theory the psychoanalytic significance of the body took on an increasingly central position (the ego, in the second topographic model, is understood primarily as a being a physical entity) its psychic processes appear coherent with the neuroscientific data on brain activity at baseline conditions (Cieri and Esposito, 2019). Furthermore, the ego, according to Freud, is made to contend with different demands, stemming both from the external reality and certain interdicts, but also with the demands of the drives coming from the body (Freud, 1923); something that further supports the parallel with the DMN.

Carhart-Harris and Friston (2010) have proposed integrating this parallelism between the DMN and the binding function of the ego in Freudian theory into the framework of the FEP. On the grounds that the DMN (like the ego according to Freud) processes and integrates the interoceptive and exteroceptive signals, one of the functions of the DMN could be the processing of endogenous excitation and the minimizing of the free energy.

It should however be underlined that drawing this parallel between the Freudian model and contemporary neuroenergetics poses an epistemological difficulty. It is true that in his *Project* (1895a) Freud posited a neuronal model of the psychic mechanism. Yet, his abandoning the publication of that manuscript indicates a renouncing of any ambitions to locate the psychic processes within anatomical structures. In chapter VII of *The Interpretation of Dreams* (Freud, 1900), he formulates this renouncement of a model based on anatomical location, thus,

I shall entirely disregard the fact that the mental apparatus with which we are here concerned is also known to us in the form of an anatomical preparation, and I shall carefully avoid the temptation to determine psychical locality in any anatomical fashion. (Freud, 1900, p. 536)

The proposal to locate the Freudian mechanism of the ego within a cerebral network is therefore to step away from the epistemological project at the origin of psychoanalysis. Nevertheless, beginning in the 1900's an important new current of research, “neuropsychoanalysis,” proposed the creation of a new discipline. The objective was to put in place an integrative approach. This approach would bring together psychoanalysis and the neurosciences within a new entity, using the methods and the techniques introduced by neuroscientific developments, such as the data from functional imaging. The aim of this new discipline was to form a link between psychic processes described by psychoanalytic theory and the neuronal structures involved in psychopathological disorders. According to Solms and Turnbull, this approach is the only one that gives scientific objectivity to psychoanalysis. This is an objectivity without which psychoanalysis consists purely in a study of subjectivity devoid of any scientific or experimental foundations (Turnbull and Solms, 2007).

The analogy between the function of the binding of the ego's psychic energy described by Freudian theory and the mechanisms of the DMN as it has been described by Friston et al. fits into this neuropsychoanalytic perspective.

In Freudian theory, the ego seeks to convert the free energy into a state of bound energy. As early as his “Project for a Scientific Psychology” (1895a) Freud had posited a main function of the psychic apparatus: the tendency to discharge quantities of excitation stemming both from within and from without the body. The distinction between free energy and bound energy, that Freud will again return to in *The Interpretation of Dreams* (1900a), encompasses in fact both primary and secondary processes that make up two levels of functioning in the psychic apparatus. This division of energy into two categories of differing nature brings to mind the first distinction made by Helmholtz between free energy (that can turn into different kinds of energy), and bound energy (that is manifest solely as calorific). However, in Freud's writings “free” should be understood as “free to move” (*frei beweglich*) and not as “free to transform” (Laplanche and Pontalis, 1988).

The free flow of the energy corresponds to the primary mechanism of the psychic functioning that Freud had initially called principle of neuronic inertia, in which the energy has a tendency to be discharged in a complete and immediate manner. The secondary process, a psychic mode of functioning that appears in a second phase, involves a binding of the energy. That is to say, the energy is pent-up and retained within certain neurons or neuronal networks, where it accumulates. Dating from 1895, it was to the ego that Freud had attributed the task of binding the psychic energy; of maintaining it constant at a relatively high or at the very least positive level, a level “above zero,” within which a constant level of cathexis is maintained. Hence, the term *Bindung* (binding) indicates at that stage the movement from a state of free energy (where it would flow through the fastest routes leading to discharge, and thus to a zero level), to one of bound energy (that is, maintained at a low but positive level, preventing the immediate flow toward discharge). According to the theory of neurons presented in his “Project for a Scientific Psychology” (1895a), the binding is made biologically possible through the existence of an network of neurons between which connections could be built: “Now the ego itself is a mass like this of neurones which hold fast to their cathexis–are, that is, in a bound state; and this, surely, can only happen as a result of the effect they have on one another” (Freud, 1895, p. 368).

This binding function of the ego is, according to Carhart-Harris and Friston (2010) consistent with the mechanisms of the DMN. A number of sources argue that one of the main functions of the DMN is the elaboration of internal mental simulations whose purpose is adaptive (Andrews-Hanna, 2012; Buckner, 2013). By integrating this observation into the FEP model, it can be deduced that the system tries to maintain the internal equilibrium of the organism through these mental simulations or representations aimed at adaptation (Carhart-Harris and Friston, 2010). Thus, the DMN, acting as a mediator between the internal and external stimuli is, from the point of view of the FEP, an essential tool for maintaining equilibrium. The energy investment by the system in trying to maintain the lowest levels of entropy (Carhart-Harris, 2018), reduces the risk of encountering the unexpected, and thus minimizes future uncertainty (Friston et al., 2012; Cieri and Esposito, 2019). In Friston's model of the FEP, each system tries to eliminate the free energy of its subordinate systems, through a process of optimisation of predictions for reducing errors. In this process it is the DMN that is most central (Carhart-Harris and Friston, 2010). The parallel between the Freudian ego and the DMN seems also to be supported by studies that demonstrate that the connectivity between the different nodes in the DMN is absent in newborns, and that it is built and matures during development (Fransson et al., 2007; Fair et al., 2008; Kelly et al., 2009). This could correspond to the Freudian hypothesis of an ontogenetic development of the ego during the primary narcissism stage (Freud, 1914). Furthermore, the hypothesis according to which one of the functions of the DMN (akin to the Freudian ego) could be the integration of interoceptive and exteroceptive signals, can also lead to one to consider that the processes of brain plasticity (by which experiences stemming from our interaction with the environment are inscribed and leave a trace in our neuronal network) contribute to the brain's expenditure in energy at its baseline condition. These processes could correspond to an “offline” mode of functioning of the brain, to the extent that they involve mechanisms that, even if they are triggered by perception, are temporally out of step with that perception. The mechanisms of plasticity can thus be deployed following a perception even when that perception has ended, since the information continues to be processed by the brain (Ansermet and Magistretti, 2010).

The mechanisms of neuronal plasticity (such as long-term potentiation and depression, or the processes of consolidation and reconsolidation, that unfold several hours to several day after the experience has taken place) call on a great many cellular and molecular processes: the activity of the ionic channels, the activation of enzymes, protein synthesis, or the regulation of the expression of different genes that contribute to the structural modification of synapses. These processes present a energetic cost to the brain, and that even when it is performing no specific task or activity (Ansermet and Magistretti, 2010). The processes of plasticity can therefore add to the significant expenditure in energy observed during the baseline state, therefore participating in the activity of the DMN.

In this way, if the mechanisms of plasticity contribute to the continued energy activity of the brain in resting state, this view encourages the drawing of a parallel between the energy consumption of the brain in default mode and the functioning of the unconscious. Indeed, in 1925, when Freud proposed a theory of the unconscious as a system of psychic traces stemming from the perceptual experiences, he had specifically pointed to the existence of such an alternating functioning of the psychic apparatus. Periods when conscious perceptual attention was directed on external environmental stimuli would be followed by periods during which this exteroceptive activity would cease. The functioning of the conscious perceptual system tasked with receiving the stimuli coming from the environment would thus be discontinuous, and the traces resulting from the periods of excitation would subsequently be preserved and stored within the unconscious. The unconscious would form, inversely to the perceptual apparatus, a system whose functioning is continuous, where traces would continuously be processed and reassociated with each other following different criteria of association (Freud, 1925). In that regard, the opposition between an unconscious system that functions continuously, and a discontinuous perceptual system focused on the environment (a system that stops during, for instance, daydreaming or sleep), can present a resemblance with the “two views of brain function” developed by Raichle. This is a view that is based on data from functional imaging, where two types of brain activity alternate: one made up of the activation of certain areas during deliberate conscious tasks, the other linked to the activity of the DMN when no perceptual or deliberate activity is taking place (Raichle, 2010).

In a more general way, it remains particularly interesting to consider the hypothesis according to which the perception of somatic states and the integration of interoceptive information combined with information coming from the environment (processed offline by the mechanisms of cerebral plasticity), could constitute one of the components of the energy expenditure of the DMN; as well as to reflect on these data in the light of FEP. This interdisciplinary perspective, aimed at bringing together research on the DMN with concepts stemming from psychoanalysis, shows significant promise with regards to its application to the understanding of certain psychiatric pathologies. Over the past few years, the DMN has received increasing attention and it has come to light that this network undergoes alterations in neurodegenerative illnesses, as well as in certain psychiatric pathologies (Cieri et al., 2020). More specifically, the data coming from neuroimaging attest to the existence of a hyperactivity of the DMN in schizophrenia. These data are particularly interesting when seen through the prism of the energetics concept of the psychic processes developed by Freud (notably his description of the libidinal processes at work in psychosis), and when we consider them within the FEP.

## The hyperactivity of the default mode network in schizophrenia

Since publication in 2001 of Raichle's first article on the baseline state *A Default Mode of Brain Function* (Raichle et al., 2001), close on 3,000 studies have been done on this subject in laboratories around the world. Among these studies, work focusing on the relationship between the functioning of the baseline state and psychiatric or neurological illnesses represent by far the largest proportion (Raichle, 2015). There exists an anomalous functional connectivity of these networks in several mental disorders, such as anxiety, depression, bipolar disorder, OCD, autism, and Alzheimer's disease (Andrews-Hanna et al., 2014). Buckner (2013) has underlined the importance of studying the DMN for psychosis, stating that “Psychosis may be a network disturbance that manifests as disordered thought, partly because it disrupts the fragile balance between the default network and competing brain systems.” As regards the study of alterations of the baseline network state in schizophrenia, many studies have highlighted the existence of a hyperactivity of the DMN in patients with schizophrenia when compared with healthy control groups. There are, however, studies in the literature that reach the opposite conclusion (Zhao et al., 2018; Lee et al., 2019). An absence of concurrence comes out of these studies. The results remain difficult to interpret, notably because of a significant variability in symptoms between individual (Lafargue, 2010).

Based on a study of MRIs focused on the baseline state in schizophrenia patients suffering from auditive hallucinations, Mallikarjun and his team have suggested that these divergences in results could be explained by differences observed between patients who have recently had their first psychotic episode and chronic patients who have been treated with neuroleptics for a number of years. In order to reduce these biases, recent studies have sought to distinguish between cohorts of patients who have recently decompensated and have thus not benefited from treatment and chronic patients who have received treatment for many years. Proposals have also been made to differentiate patients according to the nature of their symptoms. Some studies have also included, in addition to patient groups and control groups, groups of first-degree relatives of schizophrenia patients, or subjects who present an increased risk factor for schizophrenia but who have no apparent symptoms. A review of the data furnished by MRI studies of the DMN in patients with schizophrenia has highlighted that the majority of studies observe an increased functional connectivity in the brain activity in these patients–including in chronic patients and patients receiving treatment (Hu et al., 2017). However, three studies found opposite results, observing a reduction in functional connectivity in resting state (Bluhm et al., 2007; Zhao et al., 2018; Lee et al., 2019). All other studies mentioned by the review evidenced the existence of a significant hyperactivity of the DMN in schizophrenia patients (Liu et al., 2012), be it at the onset of illness or in chronic cases (Zhou et al., 2007). This hyperactivity has also been observed in individuals who present high risk factors for psychosis, as well as in the siblings of schizophrenia patients who do not themselves present signs of psychosis (Shim et al., 2010). The schizophrenia patients present an increased brain activity and an increased connectivity at resting state within the dorsolateral prefrontal cortex (Guo et al., 2017), the frontal gyrus, the insular cortex and the dorsolateral cortex, the medial and superior temporal gyrus, and the posterior cingulate cortex (Galindo et al., 2018). Their healthy siblings or close relatives present increased activity within the cingulate cortex and the anterior prefrontal cortex (Guo et al., 2017; Galindo et al., 2018).

Additionally, the schizophrenia patients also present anomalies in the usual deactivation of the DMN during the performance of tasks focused on the environment or requiring high levels of attention, when compared with the control groups (Hu et al., 2017). The activation of the attention system networks when a task where attention is focused on external stimuli is performed, usually involves a deactivation of the DMN (these two networks are termed negatively correlated). This negative correlation can be explained by a competition between the attention resources that are either focused on the exterior or the interior, depending on the need for responses to stimuli coming from the environment (Fransson, 2005). These data can, as we have seen, present some similarities with the Freudian description of the conscious perceptual system's discontinuous mode of functioning; attention being, according to Freud, only punctually outwardly focused, as though consciousness “stretches out feelers, through the medium of the system Pcpt.-Cs., toward the external world and hastily withdraws them as soon as they have sampled the excitations” (Freud, 1925, p. 231). While the rest of the time, psychic activity is dominated by internal activity during which representations stemming from mnesic traces left by the external excitations can be reassociated with each other.

The model of negative correlation between the DMN and the Task Positive Network (TPN) brought to light by recent research in neuroscience appears particularly impaired in schizophrenia patients. This is noticeable in patients suffering from paranoid schizophrenia (Zhou et al., 2007), and in those with dominant positive symptoms (Garrity et al., 2007), as well as in some first-degree relative of schizophrenia patients (Whitfield-Gabrieli et al., 2009). The functional hyperactivity of the DMN in schizophrenia patients would thus be present both at resting state as well as during the performance of tasks; and, in general, the patients whose pathological symptoms are the most significant are those who present the lowest level of deactivation of this network during tasks. According to the authors, this defect in the deactivation of the DMN during positive tasks could explain the attention and cognitive disorders observed in some schizophrenia patients (Whitfield-Gabrieli et al., 2009). Since the DMN is usually associated with the presence of thoughts independent of external stimuli–for example introspection and self-referential thoughts–it seems coherent that results, obtained during tasks that require a high level of attention focused on the environment, should be less good when the internal activity is high. This would be because the high level of internal activity could contribute to deficiencies in terms of vigilance and attention owing to competition with the self-referential cognitive activity (Whitfield-Gabrieli and Ford, 2012), for example when a significant delusional activity is present.

The significant number of studies having observed functional hyperactivity and hyperconnectivity within the different areas of the DMN (be it in resting state or during the performance of tasks) in patients with schizophrenia, has led Hu and Zong to state that this discovery represents some of the most important neurobiological data to emerge from recent research on schizophrenia:

On the basis of recent evidence, functional hyperconnectivity within the DMN is perhaps the most common finding in comparisons of schizophrenia patients with healthy controls. It has been extensively found in different schizophrenia populations, including chronic paranoid schizophrenia, first episode schizophrenia, individuals at ultra-high risk for psychosis, and the first-degree relatives of patients with schizophrenia. (Hu et al., 2017)

According to Liu and his team, the hyperactivity of the DMN may more specifically constitute an endophenotype of schizophrenia, but other members of the population could present with a hyperactivity of the DMN without presenting psychotic symptoms (Liu et al., 2012).

Furthermore, if in the majority of studies this hyperactivity is observed, these conclusions should be put into perspective by the extremely variable nature of the results. To explain these differences, some scientists have tried to establish a correlation between the nature of the symptomatology the schizophrenia patient presents with and the anomalies of activity of the DMN. The presence of auditory hallucinations (already linked to activity of the areas involved in auditory processing, language and memory, as well as the salience network) has been linked to hyperactivity of several areas of the DMN. A study by Mallikarjun has shown that the superior temporal cortex, the insula, the precuneus, the posterior cingulate cortex, and the parahippocampal region, are particularly active areas in subjects who have presented auditory hallucinations during the fMRI sequence (Mallikarjun et al., 2018). The patients who hallucinate show an increased functional connectivity at rest between parts of the salient network and the DMN, but a reduced functional connectivity between the claustrum and the insula, compared to the healthy cohort. Scientist have expressed the hypothesis that the hyperactivity observed within the DMN, and the salience network, could be linked to an alteration in the evaluation of the salience of the internal activity, an alteration that would then contribute to the origin of the hallucination.

Although the function of the claustrum remains poorly understood, some studies have brought to light that this structure may be involved in consciousness of perceptual experiences. Anomalies of the claustrum could compromise the synchronization between the associative cortex of the prefrontal lobe and the parietal lobe (Koubeissi et al., 2014; Yin et al., 2016; Fodoulian et al., 2020).

Another similar theory of the function of the claustrum posits that this brain region is involved in the timing and coordination of the cortical activity that results ultimately in perceptual experience. Disruption of the binding or synchronization of sensory, cognitive, and motor information could contribute to delusions and hallucinations in schizophrenia patients. There is a possible further link between the severity of delusions in the disease and structural abnormalities in the claustrum (Cascella and Sawa, 2014).

It has also been proposed that the diminution in functional connectivity observed between the claustrum and the insula could lead to a compensatory overactivation within some areas of the auditory network, including in some areas of the DMN and areas associated with the processing of language and memory (Mallikarjun et al., 2018).

These data regarding an increased activity of the DMN in patients who present with auditory hallucinations support the results from studies that have demonstrated that the hyperactivity in the DMN could be attributable to what is termed the “positive” symptomatology of schizophrenia (Garrity et al., 2007). It has also been demonstrated that, inversely, a correlation exists between negative symptoms and the activity of the right anterior prefrontal cortex at resting state (Reichenbach et al., 2012). The increased hyperactivity is particularly important in schizophrenia patients who present with delusional ideas with a paranoid thematic, when compared with other patients (Zhou et al., 2007) and, as we have seen, in patients suffering from auditory hallucinations (Garrity et al., 2007).

## The positive symptoms of schizophrenia according to the Freudian perspective

Within the scope of a dialogue between psychoanalysis and neuroscience centered on an energetics concept of mental activity, and in the light of a psychoanalytic understanding of psychosis, it is particularly interesting to deliberate the data coming from neuroimaging, in particular data that bring to light the existence of an increased hyperactivity of the DMN in patients with schizophrenia who present positive symptoms (characterized by delusional ideas and auditory hallucinations). To do this, we are fist going to evoke the Freudian hypotheses regarding the positive symptoms of schizophrenia, particularly from the perspective of delusions in psychosis understood as an “attempt at recovery.”

Freud, as we have seen, had proposed early on in his work a concept of mental disorders based on energetics. Once he had abandoned references to physiology and the anatomy of the nervous system, it is the concept of libido that becomes the focal point of the economics of his metapsychology. Starting with his study of the Schreber case, Freud narrowed down his understanding of the libidinal processes at work in psychosis. In the context of his disagreement with Jung, he remodeled his definition of libido. Freud's research led, in *On Narcissism: An introduction* (1914c), to the distinction between narcissistic libido which cathects the ego and an object libido turned toward the outer world. The introduction of narcissism would then enable Freud to disentangle the two phases of the psychotic process that consisted in a twofold libidinal movement. The first phase of psychosis consisted in a massive withdrawal of libidinal cathexis, of all the psychic energy, from external objects; the libido flowing back, exclusively on the ego and the subject's own body. This first phase was characterized by the fact that the patient (notably in those with schizophrenia) “…withdraws his interest from the external world completely…” (Freud, 1911, p. 75). The negative symptomatology of schizophrenia, such as being withdrawn, blunted affect, apragmatism, and also deteriorations in representations of the body (cenesthetic hallucinations, hypochondria, dysmorphophobia) are characteristic of this initial phase.

With Schreber this initial libidinal phase was expressed notably by a world's end fantasy, in which the universe is perceived by the subject to be totally devastated and depopulated, with men reduced to being only botched shadows:

The patient has withdrawn from the people in his environment and from the external world generally the libidinal cathexis which he has hitherto directed on to them. Thus everything has become indifferent and irrelevant to him… The end of the world is the projection of his internal catastrophe; his subjective world has come to an end since his withdrawal of his love from it. (Freud, 1911, p.70)

Freud postulated that this first phase is common to all forms of psychosis. However, in order to fight against this movement of libidinal withdrawal, some patients put in place a second process that would consist in an attempt to reinvest external objects with the libido. This second phase found its expression in productive symptomatology such as auditory hallucinations and delusional ideas:

When he does so replace them, the process seems to be a secondary one and to be part of an attempt at recovery, designed to lead the libido back to objects. (Freud, 1914, p. 74)

With Schreber, the paranoid delusion thus formed an attempt to “reconstruct” the world, for after having withdrawn his libidinal cathexis from the external objects of the world in the withdrawal phase, he sought to reinvest objects through his delusion. It was in effect a delusional external reality he constructed but, from a strictly libidinal perspective, this motion did constitute an attempt to reinvest an objective world: “We take to be the pathological product is in reality an attempt at recovery, a process of reconstruction.” (Freud, 1911, p. 71).

The logic of delusion as attempt at recovery should thus be understood primarily as relating to a libidinal and energetic dynamic, and in the diachronic perspective of a succession of two phases within the psychotic process. Freud's formulation therefore puts the emphasis on the essentially temporal character of psychosis. The delusional construct unfolds progressively, as Schreber's autobiography demonstrates, and can sometimes spread over an entire lifetime. In this, Freud is breaking with theories that see delusion as a deficiency; in a gesture that is seen as radically subversive by classical psychiatry. Thus, he proposes assimilating the delusion production to an “autoplastic” creative act (106), similar to an *active phase* involving a high cost in energy since through this delusion process “the ego creates, autocratically, a new external and internal world;” (Freud, 1924a, p. 151).

Furthermore, Freud argued that the process of delusional reconstruction implied not only the creation of a new reality but also required the psychotic to rebuild they memories, that is to say “…the store of memories of earlier perceptions…” (Freud, 1924a, p. 150) that constituted a copy of the past world. Thus, the reconstruction not only necessitated the generation of new current perceptions, perceptions through which “Normally, the external world governs the ego…” (Freud, 1924a, p. 150) but must also, in order to be operant, modify past perception, as though they were witnesses to a previous world, which must be erased:

In psychosis, the transforming of reality is carried out upon the psychical precipitates of former relations to it – that is, upon the memory-traces, ideas and judgements which have been previously derived from reality and by which reality was represented in the mind. (Freud, 1924b, p. 185).

The scope of the delusion reaches even into memory itself, remodeling the mnesic traces despite them being the traces of past perceptions. Now, if according to Freud the memories of these past experiences form the main basis of our relationship to the real world, this relationship is nevertheless not closed and is “…continually being enriched and altered by fresh perceptions.” (Freud, 1924b, p. 185). The unconscious work of this psychotic rebuilding therefore also has as its objective, the creation of perceptions that can correspond to a new reality, aim that would be “…most radically effected by means of hallucination.” (Freud, 1924b, p. 186).

Freud, therefore, sees auditory hallucination as being an integral part of reconstruction of the delusional world in a dialectic between memory and perception: if the delusion rebuilds the past traces of the relationship to reality through mnesic illusions of false memories, it also reconstructs the current relationship through the creation of new perception (Freud, 1924b). Auditory hallucinations should thus be understood as *mad traces* (Assoun, 2005). Indeed, for Freud, hallucination does not belong to a perception that is false or without object but is to be seen more specifically as a confusion between a memory and a current perception (Freud, 1923), where the memory finds itself invested with the strength that usually characteristic of the cathexis of external perceptions.

The mechanism through which the delusional reconstruction operates, from the Freudian perspective, generally involves a repression of all the mnesic network. The creation of a new reality does not come about *ex nihilo* but happens on the basis of mnesic traces that have really been perceived. Traces that are then reassociated and reorganized between themselves in new ways.

## Hyperactivity of the DMN in schizophrenia and the reassociation of traces during delusions

The Freudian psychoanalytic perspective according to which the positive symptomatology of psychosis (delusional ideas and auditory hallucinations) is the result of a process of trace reassociations, is interesting to put into perspective alongside the neuroimaging data that evidence a hyperactivity of the DMN in schizophrenia patients, in particular those for whom positive symptoms are predominant. Nowadays, this interaction between reassociation of traces and DMN might be accounted for within the framework of the free energy principle (FEP) (Rabeyron and Massicotte, 2020), and various forms of failure of the process of linking, regulation and transformation of free energy within the psychic apparatus could be considered as the origin of different psychopathological manifestations (Rabeyron, 2021).

As we have seen, the activation of the DMN is generally associated with spontaneous cognitive activity and self-referential processes; in particular the remembrance of autobiographical memories and projections into the future (Vincent et al., 2006; Andrews-Hanna et al., 2010; Doucet et al., 2011). The activation of the DMN in the absence of a task focused on the environment (Andrews-Hanna et al., 2010) appears to be particularly dominated by the recollection of autobiographical memories (Doucet et al., 2011). These processes, as we have seen, involve a neuronal network that belongs to the DMN, one that associates certain regions of the parietal cortex and regions of the hippocampus (Gusnard and Raichle, 2001; Vincent et al., 2006). We have seen that the medial parietal cortex, as well as the medial prefrontal cortex, are themselves the seat of hyperactivity at resting state in patients with schizophrenia. Neither do these areas deactivate, as much as in the healthy control group, during the performance of a specific task.

Thus, one can make the hypothesis that the hyperactivity of the DMN in psychosis constitutes the physiological expression of the massive reassociation of traces taking place as part of the psychotic process. This is a process that, notably, involves autobiographical memories. The participation of brain areas which are part of the DMN in autobiographical memory and in the planification of future event, two aspects which are essential to the generation self-representation (what Damasio terms autobiographical self), has encouraged scientist to make the link between changes to self-representation in schizophrenia and the presence of an excessive cerebral activity at resting state. As Lafargue underlines:

The function of the DMN is probably to ensure the coherence of thoughts throughout life, be it memories or imagining the future, by facilitating the elaboration of mental models linked to personal events. This function of cognitive oversight appears to be altered in schizophrenia. Anomalies in the functioning of the DMN have been brough to light (Lafargue, 2010, p. 47)[our translation]

If, as we have seen, the productive symptomatology may stem from a massive reassociation of traces, hyperactivity of the DMN would thus appear to be the physiological outcome of this process. The proliferation of representations at the onset of the process of reconstruction of the world performed by the delusions [prodrome that the alienists had described as an “incubation period” of the delusional ideas (Sérieux and Capgras, 1982)] might manifest itself through an increased activity of the spontaneous cognitive processes involving, and therefore an increased expenditure of energy in the areas associated with the DMN. The hyperactivity observed in the DMN in schizophrenia particularly affects the posterior regions (such as the posterior cingulate cortex and the inferior parietal cortex) and the anterior regions (medial prefrontal cortex, hippocampus and lateral temporal cortex). These are areas that are almost identical to those activated during experiments that require the subject to recall certain autobiographical memories (Svoboda et al., 2006), and encompass specifically the parietal-hippocampal network that Vincent and his team describe as particularly involved in episodic memory and the recollection of memories. The function of the delusion according to Freud focuses on “…the store of memories of earlier perceptions…” (1924b, p. 150). In order to rebuild reality, the delusional construct must, even before creating new perceptions, modify the *reflection* of the external world that are our representations stemming from mnesic traces left by past perceptions. If the *remodeling of reality* focuses selectively on “…the memory-traces, ideas and judgements which have been previously derived from reality” (Freud, 1924b, p. 185), we may conceive that, from a neurobiological perspective, this reconstruction makes significant demands on the regions of the brain involved autobiographical memory.

Furthermore, the areas situated in the medial line of the DMN are also activated when the subjects anticipate or imagine future events (Addis et al., 2007). This aspect plays a significant role in delusional constructs. Specifically, anticipation of events to come appear to be one of the major components of most delusional thematics. Delusions of persecution are characterized, in their most severe instances, by a massive anxiety on the subject's part regarding their future, even going as far as believing that an attempt on their life is imminent. With erotomania, it is generally the prospect of marriage or of imminent reunions with the loved one that dominate the representations linked to the future. This is the case with the patient Freud describes in his article *The Neuro-Psychoses of Defence* (1894a), who is convinced that her beloved, who had abandoned her, will return to her at a specific date. Similarly in the final evolution of Judge Schreber's delusion, where peace seems to come from the fact that he defers the realization of his transformation into a woman to a distant future (Schreber, 2000). Representations linked to projections into the future are thus particularly solicited by the delusional activity, and that even within the different thematics of delusion.

Finally, studies on the correlation between the different forms of spontaneous cognition and the activation of the DMN have brought to light that the temporoparietal junction, the cingulate cortex and the precuneus (areas that are particularly active at resting state in schizophrenia patients) (Saxe and Kanwisher, 2003; Spreng et al., 2009) intervene not only in autobiographical memory but are also activated when the participants try to imagine a situation through the eyes of someone else, “putting themselves in another person's shoes.” What cognitive psychology terms theory of mind–that is to say, the ability to project oneself into, and imagine what is happening in, the mind of another person. Now this cognitive process is particularly involved in the construction of delusional ideas–notably in paranoid ideas, where others are imputed with malevolent ideas.

The hypothesis of a strong involvement of spontaneous cognition–more specifically autobiographical memory, the representation of future events, and theory of mind–in the activation of the DMN, brought to light by several studies of imaging, looks to be particularly promising for explain the existence of cerebral hyperactivity at resting state in schizophrenia patients. As Bastin underlines:

In fact, all the tasks that activate the DMN require that the individual project themselves into a situation other than the reality that surrounds them. They abstract themselves from the environment to build a mental model representing alternative scenarios. Indeed, rather than process external stimuli, cognition is turned on the self, generating personal memories, imagining possible future events, and reflecting on personal emotions and motivation, as well as those of others. It is then, a question of generating mental content oneself. (Bastin, 2018) [our translation]

It is precisely this capacity to generate and construct new scenarios, separate from external reality, that is significantly mobilized in the process of delusional reconstruction such as Freud envisaged it in the form of the construction of a new reality. The “autoplastic” (Freud, 1924b) creations of psychosis–where the subject brings together reassociations of traces that form a new assembly of representations, discontinuous from the initial perceptual experiences–can thus be characterized as belonging to that type of internal cognition that is self-referential, focused on the self, and generating personal memories that are modified, reassociated, then put into perspective with representations of some future events. The hyperactivity of certain regions of the DMN observed in schizophrenia –particularly within the prefrontal cortex, the precuneus and the parietal areas –, in so far as that it affects the areas involved in episodic memory, the recollection of autobiographical memories, the representations of other people's somatic states, and the ability to generate mental simulations as well as projection into the future, could therefore be correlated with an important activity of these different cognitive functions during the process of constructing a new realty by way of delusions. This hypothesis can be underpinned by the fact that scientists have frequently noted a correlation between this hyperactivity and the presence of a positive symptomatology in the schizophrenia patients (Whitfield-Gabrieli et al., 2009).

## Discussion

We will now look at integrating this position with the FEP, which assimilates the functioning of the brain to the stationary state of a non-equilibrium system (Jarzynski, 1997) while also drawing on a Bayesian approach (Cieri et al., 2021). According to Friston (2009), the brain strives to minimize variations in free energy by maximizing evidence drawn from perceptual experiences and by reducing the element of surprise through an active role on the outside world (Ortega and Braun, 2010).

Freud speaks of an energy quantity that can be either free or bound. This is quite distinct from the formal notion of free energy of thermodynamics, statistical physics and active inference (Parr et al., 2022). However, we observe that psychoanalysis and the neurosciences agree when it comes to the observation that some representations–particularly those stemming from our experiences with the environment, which are stored in our autobiographical memory–can work as a medium for the binding function. These representations take on, in combination with traces from somatic states, a homeostatic role in the psychic balance and are generators of a form of mental equilibrium (Freud, 1900; Ansermet and Magistretti, 2004, 2010; Tran The et al., 2021).

In a FEP perspective, we can assume that the brain tries to maintain an internal equilibrium based on representations constructed from traces that stem from our past experiences. These traces are associated with our internal physiological and somatic states. As Solms has suggested, this minimizing of the free energy can be put in parallel with a homeostatic understanding of the regulation of physiological states (Solms, 2013, 2019; Solms and Friston, 2018). This is a point of view that is also held by Damasio (2000). From this standpoint the DMN, acting as mediator between the internal and external stimuli, is an essential tool for the maintaining of an equilibrium aimed at minimizing free energy (Cieri and Esposito, 2019). Specifically, the superior cortical areas try to organize the activity of the lower levels by decreasing their free energy (Carhart-Harris and Friston, 2010).

The French psychoanalyst Jacques Lacan took a particular interest in the idea that the traces that stem from the inscription of the Oedipal experience could have similar effects of balancing the psyche (Lacan, 1946). Likewise, certain traces (conscious or not) and autobiographical memories that are essential to the generation of what Damasio terms our consciousness of an autobiographical self (which are at the basis of our sense of self identity and which are stable through time), can be part of this maintenance of equilibrium in energetic terms (Damasio, 2000). This energy equilibrium might be linked to Noether's physics theorem according to which a system that has time translation symmetry is characterized by energy conservation.

It is probable that this process of equilibrium fails in the case of schizophrenia. Following the hypothesis of disconnection, this pathology is characterized by an anomalous interaction between the specialized cerebral regions. This results in a defective integration of the activity between the different networks, and thus a breakdown in cognition. With this pathology there is also a failure in the process of inference, owing to a defect in the modulation of somatic states (Friston and Frith, 1995; Friston et al., 2016). This build-up of disorder can be associated with what Freud had characterized as the primary phase of the psychopathological process at work in psychosis, a process dominated clinically by disorders in perception of the body and in interoception (Freud, 1911), which constitute the precursory stage to schizophrenia (Freud, 1915–1916/1963). This coincides with the presence in this pathology of an increased sub-cortical hyperconnectivity that translates into a heightened entropy within the brain as a whole (Salman et al., 2019). The result of psychosis would be a failure of the process of minimization of free energy and, statistically speaking, any exteroceptive or interoceptive sensation could then become surprising and unpredictable. Thus, according to Carhart-Harris and Friston (2010), in schizophrenia the functions coming down from the brain fail to predict errors. This then translates into an increase of the free energy which cannot be controlled. If we put this hypothesis alongside the role of binding free energy usually performed by certain traces and autobiographical representations, we can argue that this binding process appears to be jeopardized in schizophrenia.

This hypothesis can also be put alongside the research that has brought to light lacunae amongst the neurons involved in long-term memory and memory consolidation, in schizophrenia patients; in particular, lacunae within certain populations of neurons with precocious neurogenesis that express parvalbumin within the hippocampus (Donato et al., 2015; Genzel et al., 2015; Carvalho, 2017). These lacunae could lead to a lack of consolidation and stability in some traces and in autobiographical memories, which are essential to the generation of what Damasio terms our awareness of an autobiographical self that is at the root of our sense of self identity (Damasio, 2000). In the absence of stability over time, these traces could perhaps be more subject to lability and reassociations in psychotic patients.

Indeed, according to Bastin, the cerebral circuits of the DMN habitually “allow individuals to experience identity and consciousness” (Bastin, 2018) [our translation]. The unstable aspect of identity and of autobiographical consciousness of self in psychosis (notably because of the absence of a fixed stability of the traces that are the basis of this identity) could thus create massive functional reorganization of the DMN's activity. This neuroscientific hypothesis of a link between the hyperactivity of the DMN and a massive spontaneous cognitive activity is particularly rich in possibilities, if it is looked at through the prism of the Freudian definition of delusion as an attempt at recovery. Where the traces that make up our sense of autobiographical self usually appear to be stable and well-organized, thus guaranteeing low entropy, their lack of consolidation in schizophrenia may have as consequence a state of disorder, away from equilibrium (or from homeostasis) and an increase in free energy fluctuations. The disruption of these mnesic traces or representations that are at the root of identity and a sense of self, might then be assimilated to a dissipative process, i.e., to an internal production of entropy (Prigogine, 1967; Prigogine and Stengers, 1984). This could correspond to the presence of a globally and locally more entropic IRMf signal among schizophrenia patients. This increase in cerebral entropy constitutes an example of the disorganized peak activity that underlies schizophrenia (Sokunbi et al., 2014). According to Carhart-Harris and Friston (2010) acute psychosis corresponds to a mode of functioning described by Freud as the primary process, where the free energy is unbound. The DMN, which normally functions to minimize free energy through a hierarchy that goes from the lower to the higher levels, fails in its task. The FEP, which understands cognition as based on Bayesian hierarchical inferences and time evolution of the free energy near a stationary state, requires that the superior cortical areas work to minimize the free energy of the superior levels (Mumford, 1992; Rao and Ballard, 1999; Kiebel et al., 2008; Friston, 2010). At the onset of psychosis, the DMN appears unable to control, in a descending manner, the excess of free energy in the lower levels. More specifically, areas of the DMN such as the ACC are dysfunctional in schizophrenia, and these anomalies have also been linked to disfunctions in the anterior insula (Carter et al., 2001). Furthermore, the data from neurobiological literature on anomalies of the insular cortex in schizophrenia, have demonstrated that the disfunction of the insula can constitute one of the underlying biological causes of the disorders in corporal perception present in schizophrenia (Tran The et al., 2021).

According to the FEP, the brain uses internal hierarchical models to predict entering sensory perceptions: the brain constructs its own descending predictions (from cortex to perception) on the basis of sensory samples of the world (Friston, 2009). A failure of the integrating function of the DMN, between the exteroceptive and interoceptive stimuli, can create an inability to construct adequate descending predictions. Thus, the aim of the process is to optimize the explanation of the causes of sensory inputs, in order to establish new predictions that inform action and behavior (Friston, 2003). In schizophrenia, this process fails, and surprise (and thus the fluctuations of the free energy) will increase.

Following the Freudian hypothesis of delusions as attempts at recovery, the process of systemising the delusions can constitute an attempt by the organism to try and minimize the free energy and the disorder present in schizophrenia. This would be an instance of the tendency of all organized systems, and notably living organisms, to seek to lower the level of surprise as it interacts with its environment (Friston et al., 2015). The representations fixed by the systematization of delusions could represent an attempt to minimize the free energy, of binding it through the consolidation of the delusional representations in a process similar to that which Hopkins (2016) describes. This is a process based on the normal function of dreams, which perform a reduction of complexity (a process of binding) through the consolidation and reconsolidation of memory. Since these mechanism for reducing complexity appear to play an important role in mental disorders (Hopkins, 2016), it can be assumed that delusions are an attempt at converting free energy into bound energy through the production and the consolidation of new mnesic traces or representations. These traces or representations stem from the processes of reassociation described by Freud. According to the Bayesian approach, the systematizing of delusions generates new predictions aimed at reducing surprise and re-establishing a form of descending causality. This process echoes Freud's notions of free vs. bound energy. It is consistent also with the notion of an allostatic equilibrium of psyche.

This bringing together of the Freudian idea of delusion as attempt at recovery and Friston's FEP has the advantage not to reduce the neurobiological processes at work in psychosis to a deficit but rather as a functional reorganization. Indeed, even studies that have brought to light the existence of some deficits in anatomical connections in regions of the cerebral cortex among patients with schizophrenia argue that the functional hyperconnectivity between these regions could represent a neuronal response to that an anatomical deficit (Skudlarski et al., 2010). The dialogue between Freudian theory and neuroenergetics, therefore, reintroduces a dynamic perspective into the neurobiological study of the physiopathology of schizophrenia. This goes beyond an exclusively localizationist perspective reduced to a strictly anatomical view.

To envisage that the formation of delusional traces and representations, and the systematization of delusion, might be attempts to restore a homeostatic equilibrium by minimizing the free energy, opens up interesting new therapeutic horizons for the psychotherapeutic treatment of schizophrenia. The observation that there exists a defect in the consolidation of long-term memory in this pathology (Donato et al., 2015; Genzel et al., 2015; Carvalho, 2017), might suggest that the psychotherapeutic work with psychotic patients could have for its objective an alternative process to the delusion's function of binding free energy. The aim would be that the psychic treatment based on talking would enable a reinscription or a reconsolidation of those traces. This would take place through a process of remembering of traces linked to the patient's experiences and their personal history. The hope would be that this would stabilize elements of the autobiographical memory, which could contribute to a greater “sense of self identity,” as suggested by Damasio (2000). One could imagine that regular and repeated psychotherapy sessions could stimulate and stabilize this sense of identity and consciousness of self. The consolidation of autobiographical traces would operate a kind of binding of the free energy (or minimizing of the errors in prediction according to FEP), and a regulation of the alteration in the perception of somatic states present in psychosis.

## Conclusion

To conclude, we can say that psychoanalysis and neuroscience can find a point for discussion around the inference models of mental activity expressed in terms of a free energy principle, and that this dialogue may be particularly fruitful when it comes to the understanding of some psychiatric pathologies. We have observed that the Freudian understanding of psychosis is framed by a theory of libido and, thus, of the energetics of the psychic processes. In particular, the hypothesis of delusion as an attempt to heal involves understanding psychosis as a libidinal (therefore energetic) and diachronic process in two phases. Furthermore, Freud conceived the genesis of delusional ideas in terms of a process of “reconstruction” of reality. This is to be understood as a process that operates through the formation of new traces by means of a massive reassociation of traces stemming from past perceptual experiences. This process then introduces a significant discontinuity between the initial experiences and the new traces (delusional ideas and hallucinations) produced by the individual.

Furthermore, much of the data highlight a hyperactivity of the DMN in schizophrenia, particularly for patients in whom the positive symptoms dominate the clinical picture. In so far as studies have shown that the activation of the DMN is more specifically linked to processes of spontaneous cognition and self-referential thoughts (such as recalling autobiographical memories or as projections into the future) this hyperactivity of the DMN can be put in parallel with the Freudian concept of positive symptoms in psychosis.

In the perspective of a dialogue between psychoanalysis and neuroscience, we have thus proposed to consider the data showing a hyperactivity in the DMN in resting state in patients with schizophrenia as being correlative to a massive process of reassociation of mnesic traces. This is a process that would result in the formation by the psychotic individual of new assemblies of traces and representations, from which delusional ideas or auditory hallucinations would originate. This production of new assemblies of traces would involve those areas, termed “spontaneous cognition,” that are associated with the activity of the DMN, in particular autobiographical memory but also anticipation of future events. Indeed, the process of delusional constructions bears on the one hand on the traces resulting from past perceptual experiences, which it tries to remodel; on the other hand, it results in the generation of new representations of the individual's future. In addition, this production of new representations introduces a reconfiguration not only of the representation of the self but also representations of others and their intentions. This also joins the imaging data relating to the contribution of mental activity linked to theory of mind, on the activation of the DMN.

Following the FEP model, it is possible to envisage a function of the DMN that aims to minimize the free energy, drawing a parallel with the binding function of the ego in Freudian theory. The creation and consolidation of new representations during the process of delusional systemisation could, in that case, be thought of as an attempt at minimizing the excess of free energy present in schizophrenia. This hypothesis is in keeping with the Freudian concept of delusion in psychosis as an attempt at recovery.

If an interdisciplinary dialogue has been shown to be rich in material for better understating the symptoms and psychopathology of certain psychiatric illnesses, the data regarding the hyperactivity of the DMN still need to be better understood. Indeed, the functions of the DMN are still subject to debate in the scientific community, and some studies done with schizophrenia patients have presented sometimes contradictory results. Nevertheless, integrating the ideas stemming from psychoanalysis within the neuroscience research on schizophrenia may help understand psychosis as a diachronic process. This, in turn, could encourage the setting up of longitudinal studies to better understand the outcomes and evolution of the clinical picture for long-term patients. The Freudian understanding of psychosis in particular emphasizes the temporal and diachronic logic of this pathology, in the evolution of its libidinal–and thus energetic–dynamic. Integrating this view with research in neuroscience can thus lead to encouraging the development of longitudinal studies in imaging, in order to bring to light the evolution of the activation and the functional connectivity of the DMN at different stages of illness. If, in order to avoid biases (linked for example to the administering neuroleptic treatments to patients over several years), different studies apply protocols involving cohorts of untreated patients who have recently decompensated, it could be still more informative to be able to repeat the study of imaging on these patients at different stages of their treatment. This, in order to determine if their clinical evolution correlates with variations in the level of activity and connectivity of the brain's DMN. Performing such longitudinal studies could fit in with the essentially diachronic character of the psychotic process as evidenced by Freud in his concept of the twofold process at work in psychosis.

In their review of the neuroscientific literature relating to the activity of the DMN in schizophrenia, Hu and his team specifically encouraged the development of such longitudinal studies:

…because the divergence of findings among studies could represent differences in the activity and connectivity of the DMN that might be involved in different stages of this disease, it could be important to explore the DMN throughout the course of schizophrenia… (Hu et al., 2017)

The study of the evolution in levels of baseline brain activity in longitudinal neuroimaging protocols on psychotic patients over several years, from the onset of psychosis up to an eventual clinical stabilization, could represent a new research route. This is a route that would make it possible to take into consideration the psychotic process in its temporal and diachronic dimension, eventually perhaps bringing to light correlations between the levels of activity in the DMN and the patients' clinical pictures.

## Author contributions

JT is the main contributor of this paper as part of her postdoctoral work. FA and PM as supervisors, contributed to the conception and development of the research, and they revised critically the manuscript for intellectual content. All authors contributed to the article and approved the submitted version.
